# Draft Genome of a Novel *Chlorobi* Member Assembled by Tetranucleotide Binning of a Hot Spring Metagenome

**DOI:** 10.1128/genomeA.00897-14

**Published:** 2014-09-11

**Authors:** Blake W. Stamps, Frank A. Corsetti, John R. Spear, Bradley S. Stevenson

**Affiliations:** a Department of Microbiology and Plant Biology, University of Oklahoma, Norman, Oklahoma, USA; b Department of Earth Sciences, University of Southern California, Los Angeles, California, USA; c Department of Civil and Environmental Engineering, Colorado School of Mines, Golden, Colorado, USA

## Abstract

The genome of a member of the phylum *Chlorobi* was assembled from a shotgun metagenomic sequence of a hot spring in Mammoth Lakes, CA. This organism appears to be a novel, aerobic, photosynthetic *Chlorobi* member, expanding the knowledge of this underrepresented phylum.

## GENOME ANNOUNCEMENT

Relatively few members of the phylum *Chlorobi* have been cultivated and characterized. Most described members of the *Chlorobi* are anoxygenic photoautotrophic bacteria that occur in aquatic sediments, sulfur springs, and hot springs, reducing sulfur compounds instead of oxygen (1). Aerobic members of the *Chlorobi* have recently been described, but only through metagenomic approaches (2).

Samples were collected from one of a series of hot springs at the headwaters of Little Hot Creek located in the Long Valley Caldera near Mammoth Lake, CA (3). The springs are supersaturated for carbonate, circumneutral in pH, and range from a temperature of 50°C to 80°C. Genomic DNA was isolated from a thick biofilm growing on the surface of a carbonate structure in the spring LHC1 using the Zymo Xpedition Soil/Fecal kit (Zymo Research Corp., Irvine, CA). Sequencing was conducted using a 400 bp insert library prepared with the Agilent SureSelect kit (Agilent Technologies, Irvine, CA) and Illumina MiSeq v4 PE300 sequencing. Raw reads were first assembled in CLC Genomics Workbench 7.0 (CLC Bio), and then the scaffolded contigs were binned by tetranucleotide frequency using Metawatt (4). Post binning raw reads were remapped to the binned scaffolds to establish the sequencing coverage and relative abundance of the genome within the metagenomic sequence.

The NCBI Prokaryotic Genome Annotation pipeline was used for genome annotation. The genome assembled into 51 scaffolds over 3.06 Mbp and contained 2,918 coding regions and 45 RNAs. The 16S rRNA gene showed a low similarity (91%) to currently characterized members of the genus *Chlorobium*. This draft genome, therefore, represents a potentially novel genus within the *Chlorobi*. The genome comprised 5.2% of all reads sequenced from the environmental sample, and a G+C content of 50.4%. The annotated genome contains an *fmo* (bacteriochlorophyll A) gene with high similarity to other members of the genus *Chlorobium*. In addition, other genes associated with the production of bacteriochlorophyll and chlorosomes common to the *Chlorobi* were also present. The annotation of this binned genome suggests that the representative population is unable to oxidize sulfur and is potentially aerobic. This is similar to another recent environmental isolate of the *Chlorobi* (2). The genome sequenced represents a potentially deep branching member of the *Chlorobi* and provides crucial data to this underrepresented group of microorganisms.

### Nucleotide sequence accession number.

This whole-genome shotgun sequencing project has been deposited at GenBank under the accession number JPGV00000000.
